# Preliminary Insights into the Diagnostic Accuracy of the Modified Arm Care Screen Test for Overhead Athletes: An On-Field Tool for Injury Prevention

**DOI:** 10.3390/healthcare11233046

**Published:** 2023-11-26

**Authors:** Eleftherios Paraskevopoulos, Fotis-Marios Kottaridis, Maria Moutzouri, George A. Koumantakis, Dimitrios Antonakis-Karamintzas, Charilaos Tsolakis, Panagiotis Koulouvaris, Anna Christakou, Maria Papandreou

**Affiliations:** 1 Department of Physiotherapy, University of West Attica, 12243 Athens, Greece; moutzouri@uniwa.gr (M.M.); gkoumantakis@uniwa.gr (G.A.K.); mpapand@uniwa.gr (M.P.); 2 Department of Physiotherapy, University of Peloponnese, 23100 Sparta, Greece; pth19057@uop.gr (F.-M.K.); a.christakou@go.uop.gr (A.C.); 3 1st Department of Orthopaedic Surgery, School of Medicine, National and Kapodistrian University of Athens, 12462 Athens, Greece; dkaramintzas@med.uoa.gr (D.A.-K.); tsolakis@phed.uoa.gr (C.T.); info@drkoulouvaris.gr (P.K.); 4 Sports Performance Laboratory, School of Physical Education & Sports Science, National and Kapodistrian University of Athens, 17237 Athens, Greece

**Keywords:** prevention, overhead athletes, shoulder injury, balance, screening

## Abstract

Background: The Arm Care Screen was developed to detect musculoskeletal limitations that could affect performance or even increase the risk for shoulder injuries in overhead athletes. This study aimed to assess the discriminant validity of the modified Arm Care Screen in overhead athletes. Methods: Sixty-two overhead athletes (mean age: 24.5 ± 4.2 years) were recruited. The athletes underwent a comprehensive Arm Care Screen evaluation, including reciprocal shoulder mobility, total body rotation, lower body diagonal reach, and rotary stability assessments. Ten different musculoskeletal measurements were independently measured by two physical therapists. Results: The modified Arm Care Screen showed moderate to strong associations (phi values ranged from 0.273 to 0.905) with the respective musculoskeletal range of motion and balance measurements. Sensitivity ranged from 81.25% to 88.57%, indicating a high true positive rate, and specificity ranged from 43.75% to 94.44%, indicating a moderate to strong positive rate. Positive and negative likelihood ratios ranged from 1.48 to 15.92 and 0.12 to 0.38, respectively. The positive and negative predictive value ranged from 58.14% to 92% and from 73.68% to 93.18%, respectively. The accuracy of the modified ACS ranged from 62.90% to 91.94%. Conclusion: The modified Arm Care Screen demonstrated promising diagnostic accuracy in identifying significant movement restrictions.

## 1. Introduction

Overhead athletes, such as baseball pitchers, tennis players, and volleyball spikers, subject their shoulders and arms to significant stress during repetitive and forceful movements [1]. Previous research has observed a 40% prevalence of shoulder pain related to overuse pathology [2]. Maintaining optimal shoulder and arm function is not only essential for peak athletic performance but also critical for injury prevention and career longevity [3]. Although guidelines have been established, a significant portion of arm injuries in overhead sports result from non-traumatic musculoskeletal issues caused by overuse [4]. In an effort to reduce the occurrence of these injuries, coaches have introduced arm care programs focused on enhancing muscle and joint strength, dynamic stability, and range of motion (ROM) [5]. These programs aim to address musculoskeletal injury risk factors and promote injury prevention among players [4,6]. Nonetheless, these arm care programs often lack specificity since coaches without access to an athletic trainer or physical therapist (PT) find it challenging to identify individual injury risk factors for each player [7,8].

Recent studies have introduced simple on-field assessment tests suitable for use by less experienced personnel and coaches without clinical backgrounds. These tests are applicable to a wide range of overhead athletes [8]. These assessments should encompass different components of the kinetic chain known to be involved in shoulder injury occurrences [4]. Research has shown that musculoskeletal risk factors for shoulder injury include a glenohumeral internal rotation deficit (GIRD) [9,10], restricted hip internal rotation (IR) [11] and external rotation (ER) [12], decreased mobility in the thoracic spine [13], asymmetry in total shoulder range of motion (TROM) [10], and compromised dynamic single-leg balance [14].

Considering the risk factors mentioned earlier, a screening tool called the Arm Care Screen (ACS) has been implemented (based on the principles from Functional Movement Systems (FMS)) to rapidly and effortlessly detect baseball players who have a heightened probability of sustaining shoulder injuries [8]. The reliability of the ACS has been examined among high school baseball coaches, with excellent intra-rater (k = 0.76; 95% CI 0.54–0.95) and inter-rater (k = 0.89; 95% CI 0.77–0.99) agreement levels [15]. Prior studies have indicated that the ACS is capable of effectively distinguishing between baseball players who possess musculoskeletal risk factors for shoulder injury and those who do not [8]. Importantly, since the ACS is based on FMS principles, it holds promise for identifying shoulder injury risk in other overhead athletes as well. However, there is currently a lack of research exploring the discriminant validity of the ACS in other overhead sports.

This gap in knowledge is significant, given the high rates of shoulder injuries identified in various overhead sports, such as volleyball, handball, water polo, and swimming [16]. Core and shoulder stability play a crucial role in the kinetic chain, potentially making athletes more susceptible to injuries and making it imperative to develop testing methods capable of evaluating these aspects [17,18].

Therefore, the objective of this study was to evaluate the discriminant validity of the modified ACS in identifying common musculoskeletal risk factors in overhead athletes. We hypothesised that suboptimal performance on the ACS subtests will demonstrate high sensitivity in detecting the presence of at least one associated musculoskeletal risk factor among overhead athletes.

## 2. Materials and Methods

A prospective cross-sectional approach was employed to determine the effectiveness of the ACS in differentiating the existence of musculoskeletal risk factors within a group of overhead athletes. The study adhered to the Standards for Reporting Diagnostic Accuracy Studies (STARD) guidelines, ensuring standardised and comprehensive reporting for the diagnostic accuracy study design. Ethical approval was received from the University of West Attica (approval number: 14679/14 February 2023) prior to data collection. All athletes consented to participate in the study after they were informed about the procedures and the aims of our research.

### 2.1. Sample

To determine an appropriate sample size, based on previous research studies, we assumed a prevalence rate of 70% for the risk factor of participant injury [8,19]. To achieve a sensitivity of 0.90 on the ACS with a confidence interval (CI) width of 0.10, a minimum sample size of 50 participants was required [8,19]. To be eligible to participate, athletes had to meet the following criteria: to engage in overhead sports (tennis, volleyball, baseball softball, water polo, handball, badminton, basketball, squash, swimming), to be active athletes aged >18 years who are asymptomatic and have no history of shoulder surgery or any shoulder-related treatments. We defined asymptomatic based on our previous research studies [20,21]. Exclusion criteria encompassed any previous occurrence of shoulder pain leading to loss of training or abstaining from active participation for more than 3 consecutive days, as well as a history of a previous shoulder injury or receiving physical therapy for the chest, shoulder, or neck. Overall, 62 athletes met the inclusion criteria and were included in the study.

### 2.2. Modified ACS

In order to assess all athletes, we attended team trainings after agreement with the coaching staff. The procedures for data collections occurred prior to their training to reduce the chance of training injuries or symptoms due to delayed muscle onset. Data collection took place over a period of 6 months, involving athletes before the start of the preparation phase (training cycle) using two independent assessors trained in the use of the modified ACS. Before the assessment, the athletes’ demographic information was recorded.

**Figure 1:**
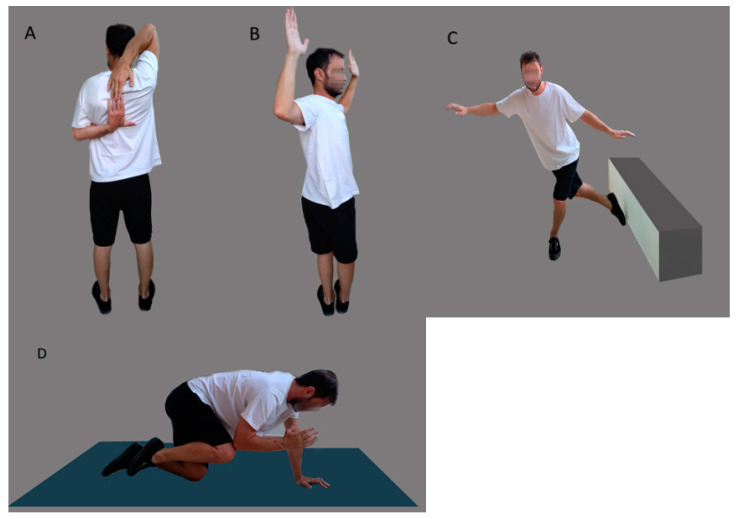
Modified ACS: (**A**) reciprocal shoulder mobility, (**B**) 90/90 total body rotation, (**C**) lower body diagonal reach, and (**D**) rotary stability.

The original ACS consists of three subtests: (1) Reciprocal shoulder mobility, where the athlete stands with feet together, reaching one hand behind the head and the other hand behind and up the back simultaneously, aiming to touch both fingertips while staying upright. Inability to do so on one or both sides is considered a positive result. (2) 90/90 total body rotation involves standing with feet together and arms in a 90/90 position, then rotating the entire body, including hips, shoulders, and head, as far as possible while keeping feet pointing straight ahead. A positive outcome occurs when the front of the opposite shoulder becomes invisible while maintaining good posture on either side. (3) Lower-body diagonal reach is performed by standing two shoe lengths away from a wall, maintaining single-leg balance while reaching the opposite leg behind and across to touch the toes to the wall three times without touching the ground for practice. The athlete then repeats the test by touching the toes to the wall five times without losing balance. Failing to touch the wall five consecutive times without losing balance is considered a positive result (see Figure 1). This test was based on the main test components of the Y-Balance test [22].

We opted to add an additional evaluation focusing on core stability (rotary stability assessment) that followed the Functional Movement Screen (FMS) guidelines [23]. In this test, the athlete assumes a hands-and-knees position with arms and thighs vertically aligned. The feet should also be vertical with the toes resting on the floor. The athlete ensures that the thumbs, knees, and toes remain in contact with the ground. During the test, the athlete simultaneously extends the right arm forward and the right leg backward, similar to a unilateral superman position. Then, the athlete retracts the elbow and right knee until they touch directly over the ground (Figure 1).

### 2.3. Risk Factors

In order for the ACS to detect musculoskeletal risk factors, precise cut-off thresholds had to be defined for each risk factor. Impairments falling below the established cut-off values for physical impairments were categorised as either present or absent based on the existing literature and the values provided in previous research, as shown in Table 1. These values were also used in the study by Matsel, Hoch, Butler, Westgate, Malone, and Uhl [8]. Two physical therapists independently measured these ten musculoskeletal risk factors separate from the ACS scoring process. Nine of these factors were reported in the study by Matsel, Hoch, Butler, Westgate, Malone, and Uhl [8]. The added risk factor was the Closed Kinetic Chain Upper Extremity Stability (CKCUES) test [17]. The procedures for the CKCUES have been described previously [24]. If an athlete scored below the threshold values reported in the literature (18.5 touches for males and 20.5 touches for females from a modified position) we considered this as an existing risk factor. Although a recent study showed that athletes that score less than 21 touches are 18 times more likely to get injured [25], we decided to use similar reference values that provided different cut-off scores for men and women (18.5 touches for males and 20.5 touches for females from a modified position) based on an older study [26]. All the risk factors with the measurement procedures, along with the tests of the ACS that we compared them with, are described in detail in Table 1.

To test the validity of the ACS, we compared the findings of each of the four assessments of the ACS (pass/fail) with the risk factor measurements (pass: above the cut-off values and fail: below the cut-off values). To establish the diagnostic ability of the ACS (sensitivity, specificity, positive predictive value (PPV), negative predictive value (NPV), likelihood ratios (LRs)) we considered the following: A true positive (TP) was identified when there was at least one shoulder or thoracic mobility risk factor and this risk factor was accurately detected by a positive result in the ACS reciprocal shoulder mobility test on either side. A true negative (TN) was recorded when the participant successfully passed all the goniometric shoulder and thoracic range of motion (ROM) tests and received a negative result on the reciprocal shoulder mobility screen for both sides. A false positive (FP) occurred when no shoulder or thoracic risk factors were present but the participant tested positive on either side of the reciprocal shoulder mobility test. A false negative (FN) was observed when a participant had at least one shoulder or thoracic risk factor but tested negative on the reciprocal shoulder mobility screen for both sides. A similar procedure was followed for the other three tests within the ACS (total body rotation, lower-body diagonal reach, rotary stability) as outlined in Table 1. Lastly, we aimed to determine the level of agreement between the two physical therapists (inter-rater reliability).

### 2.4. Statistical Analysis

Demographics were analysed using descriptive statistics. The existence of any corresponding musculoskeletal risk factor was categorised as previously described and recorded in individual 2 × 2 tables for each of the 4 components of the modified ACS. Chi-squared tests were performed to identify any associations between each component of the modified ACS and the risk factors. In case one or more expected cell frequencies were found to be less than five, the Fisher’s exact test was used [36]. The strength of the association was examined through the phi (Φ) value, as previously suggested [37]. Sensitivity, specificity, positive predictive value (PPV), negative predictive value (NPV), likelihood ratios (LRs), and odds ratios were calculated as described in previous research [38,39]. Cohen’s κ was run to determine the level of agreement between the two physical therapists (inter-rater reliability). The k value was interpreted as follows: κ < 0.20 = poor, κ: 0.21–0.40 = poor to fair, κ: 0.41–0.60 = moderate, κ: 0.61–0.80 = substantial, κ: 0.81–1.0 = excellent [23]. All statistical analyses were performed using SPSS (IBM, version 25). A significance level of *p* < 0.05 was considered statistically significant for all conducted tests.

## 3. Results

Demographics of all 62 athletes were calculated and presented in Table 2. Chi squared tests showed significant associations between each component of the modified ACS and the respective risk factors. The strength of the association (phi (Φ) value) ranged from 0.273–0.905, indicating a moderate to strong association (Table 3). Table 3 presents the precision of each element in the modified ACS when distinguishing between individuals with musculoskeletal risk factors.

The sensitivity ranged from 81.25 to 88.57 among the four components of the modified ACS, demonstrating a high true positive rate, whereas a broader range of specificity values were found that ranged from 43.75 to 94.44, demonstrating a moderate to strong positive rate. Positive LRs indicate the fold increase in the odds of having a particular condition in a participant with a positive test result [40]. This value ranged from 1.48 to 15.92. Negative LRs indicate the fold decrease in the odds of having a particular condition in a participant with a negative test result [40]. This value ranged from 0.12 to 0.38. The PPV indicates the probability of the pathology in subjects that test positive and this value ranged from 58.14 to 92%, whereas the NPV, which is the opposite in subjects that test negative, ranged from 73.68 to 93.18% (Table 4).

The accuracy value indicated the overall probability of correct classification of each athlete. This value ranged from 91.94 to 62.90 in the four components of the modified ACS. The prevalence of existing musculoskeletal risk factors varied between 25.81 to 56.45%. Finally, athletes with pre-existing risk factors experienced significantly increased odds of failing various tests. Specifically, they had 3.88 times higher odds (95% CI 1.186 to 12.74) of failing the reciprocal shoulder mobility screen, 130.3 times higher odds (95% CI 20.171–842.128) of failing the total body rotation screen, 48.87 times higher odds (95% CI 11.086–215.461) of failing the lower body diagonal reach, and 35.53 times higher odds (95% CI 7.45–169.33) of failing the rotary stability test compared with athletes who did not have risk factors, as presented in Table 4. The level of agreement between the two physical therapists was excellent, as shown in Table 5. Moreover, we also performed subgroup analysis of the aforementioned values separately for male and female athletes for each sport (volleyball, basketball, tennis) and for experienced and non-experienced athletes (Supplementary Materials).

## 4. Discussion

The main aim of this study was to assess the discriminant validity of the modified ACS. This was achieved by categorising musculoskeletal risk factors into two groups: present or absent, using a predetermined cut-off value. Subsequently, participants were classified as either passing or failing the corresponding ACS component based on this categorization. The statistical analysis provided important diagnostic values related to the sensitivity, specificity, positive predictive value (PPV), negative predictive value (NPV), likelihood ratios (LRs) and odds ratios. The findings of this study supported our main hypothesis that the modified ACS would adequately identify overhead athletes with a musculoskeletal risk factor and showed that it is a promising tool for shoulder injury prevention in overhead athletes. Moreover, subgroup analysis revealed the consistency of our findings separately for each subgroup based on gender, sport, and experience.

This research confirmed the findings of previous research about the discriminant validity of the ACS that was conducted in baseball players [8]. Due to the fact that all overhead athletes have an increased risk of shoulder injury [3], we believed that it would be valuable to test the ACS in other overhead athletes as well. This study further increased the applicability of the ACS by incorporating an additional subtest (rotary stability), which seems to be essential for core stability assessment [23]. Since core stability can influence the transfer of ground reaction forces through the kinetic chain and increase the risk of shoulder injury in overhead athletes [20,41], it seems essential for a screening tool for overhead athletes, such as the ACS, to include a relevant assessment. Although the rotary stability test was only assessed against a single risk factor (CKCUES), its performance demonstrated significant sensitivity (0.81) and specificity (0.89) values. This underscores its significant potential as a valuable tool in the realm of injury prevention. Finally, it has the capability to serve efficiently as a substitute for the laborious CKCUES, thereby saving time in the assessment process.

Shoulder ROM assessment has been used to assess the risk of injury for overhead athletes multiple times in the past [27,42,43,44]. Moreover, the Bern Consensus Statement concluded that examination of shoulder ROM should be included as part of a proper injury-prevention strategy [45]. Previous research has shown that limited internal rotation may increase the risk of shoulder pain in many overhead athletes such as volleyball players [46], tennis players [47], water polo players [48], and baseball players [27]. The explanation for this phenomenon may be related to the continuously emphasising restriction of internal rotation in the glenohumeral joint that may result in the tightening of the rotator cuff within the posterior capsule [48]. Additionally, repetitive eccentric stresses on the rotator cuff due to excessive external rotation and eccentric loading can lead to microtrauma in the rotator cuff tendons, potentially causing overuse injuries [49]. Although internal rotation reduction has been defined as a risk factor for future injury on its own, the majority of the available studies have examined the internal rotation deficit in comparison with the other limb, which is known as GIRD, and have shown that GIRD is a significant risk factor for future injury in a range of overhead athletes of various ages [44,47].

A variation in internal rotation between the dominant and non-dominant shoulders, known as GIRD of the dominant shoulder, can impact shoulder stability [44,47]. This discrepancy may potentially lead to rotator cuff impingement and labrum tears [47]. Unlike internal rotation limitation only, GIRD has been used extensively in numerous overhead athletes and has shown that a GIRD of 20° or more increases the risk of shoulder injury incrementally [47,50,51,52]. However, various GIRD values have been found to increase the risk of injury that start from only 7.5° in youth female handball players [53] and increase in other populations. For example, GIRDs of ≥10° in handball and volleyball male players [54], ≥14 in badminton players [52], and ≥18–20 in baseball [10,28] and tennis players [55] can increase the risk of shoulder injury. It remains questionable whether the modified ACS would have shown the same diagnostic accuracy for smaller GIRD values.

Total range of motion (TROM) has been defined as the sum of total external and internal rotation [56]. Research has shown that even a small difference of 5° or more between the dominant and non-dominant shoulder can increase the risk of shoulder injury in baseball and tennis [42,57], water polo [43], volleyball [46], and in some cases in handball [58]. As in GIRD, we decided to use the larger TROM difference reported as a risk factor (>10), as previously conducted [8], since this study recruited a variety of overhead athletes.

Shoulder flexion deficits of more than 5° have also shown to be related to an increased elbow and shoulder injury risk [30,59]. However, this finding has been documented in a significantly smaller number of studies and populations (baseball players only) [30,59]. It has been postulated that the relationship observed between shoulder flexion deficit and upper limb injury could indicate a deficiency in tissue mobility and general flexibility (potentially in the latissimus dorsi) among athletes prone to injuries [30]. This finding may also indicate a contribution to scapular dyskinesis, which has also been described as a risk factor for shoulder injury in the majority of athletes competing in overhead sports [30,58,60]. More research should be conducted in other overhead athletes to ensure that flexion deficits are associated with shoulder injury in overhead sports.

Similarly, with shoulder flexion deficit, trunk rotation has also been shown to be a risk factor for shoulder injury [31]; however, there is a notable scarcity of research conducted in this particular area. Although the deficits in trunk rotation have been implicated for the risk of injury in overhead athletes (baseball [31] and swimming [61]), more research is needed in other overhead sports to identify the link between trunk rotation deficit and shoulder injury risk.

The reciprocal shoulder mobility test is an easy-to-use test that can combine several movements of the shoulder and trunk. This study found that this test has the potential to identify athletes with restricted shoulder or trunk mobility in a simple assessment, since the sensitivity was high (0.83). However, it may be less specific, since the specificity was moderate (0.43), indicating that the ability of the reciprocal shoulder mobility test to identify participants with no risk factors may be limited. This is in line with the findings of the founders of the original ACS [8]. In their study, the specificity was 0.57 in comparison with the higher sensitivity (0.98). The aforementioned findings suggest that research in a larger sample of athletes may shed light on the important role of this test in injury prevention.

The lack of hip rotation has been also implicated in shoulder injury in overhead athletes [11,12,33]. Furthermore, adequate hip ROM can improve performance and reduce the risk of back pain in javelin throwers and racquetball, tennis, squash, and golf players [62,63,64]. The inter-relation between the shoulder and the hip has been examined previously [65]. It is proposed that repetitive forces during play at the hip may result in capsular contractures that reduce hip rotation [63]. The lack of hip mobility in the dominant side during throwing may increase the need for shoulder external rotation in an attempt to improve performance, which may increase the amount of stress placed on the shoulder [65]. However, more research is needed in other overhead sports to identify the cut-off values for an increased risk of shoulder injury.

Again, the total body rotation test can be performed and assessed by coaches with limited knowledge in healthcare assessment. This test demonstrated very high values of sensitivity (0.88) and specificity (0.94), increasing the valuable role of the modified ACS in injury prevention. Although this test was compared with only two risk factors (hip internal and external rotation limitation), its discriminant validity was excellent.

Reduced balance measured through Y-Balance has shown to increase the risk of injury in a range of sports including ice hockey, baseball, handball, soccer, alpine skiing, basketball, and floorball [34,35,66]. It has been suggested that the act of throwing requires a seamless coordination of balance and precision to effectively transfer energy from the lower body and trunk to the upper extremity, ultimately resulting in the release of the ball [20,66]. It is hypothesised that any disturbance in balance or the inability to stabilise the body over a supporting base might hinder muscle activation and hinder the attainment of optimal force generation. Consequently, dysfunction in the lower segments of the body could have adverse effects on an athlete’s throwing mechanics, potentially elevating the risk of injury overall [20,66].

The lower-body diagonal reach demonstrated very good reliability (0.88) and validity (0.85) values. Although research on injury risk based on dynamic balance assessment is limited, it appears that lower-body diagonal reach could be a valuable test in injury prevention. Nevertheless, further research is required, particularly among overhead athletes, to determine its association with injury for this specific group.

Although the findings are promising, this study is subject to certain limitations. The cross-sectional design employed in this research hinders the establishment of causality, and it is imperative that future longitudinal studies should be conducted to validate the modified ACS’s predictive capabilities concerning actual injury occurrence or performance improvement. Moreover, the inclusion of athletes with varying levels of experience and different positions has increased the heterogeneity of our sample, making it challenging to establish specific cut-off points for all athletes with regard to injury risk factors.

Furthermore, in our assessment of the discriminant validity of the reciprocal shoulder mobility screen subtest, we conducted evaluations of various musculoskeletal risk factors, including GIRD, TROM, and deficits in shoulder flexion range of motion (ROM) or thoracic spine rotation ROM. This approach increases the likelihood of detecting at least one risk factor compared with the other subtests of the modified ACS. Moreover, this study recruited younger athletes in their twenties. Thus, the findings of this study cannot be generalised to older (>35 years) competitive overhead athletes.

It is essential to recognise that injury risk is multifactorial, encompassing factors such as psychological status and exhaustion. The primary goal of the ACS is to heighten coaches’ awareness, particularly those working with numerous players annually, so that they can prioritise certain aspects in their exercise programs over others. These limitations are more closely tied to shortcomings that could impact sports participation and performance rather than directly influencing injury occurrence. It is crucial to emphasise that the ACS should not replace clinical assessment but can be used solely for screening purposes.

In this study we calculated nine different measures of diagnostic accuracy and one related to the levels of agreement between the two raters when using the modified ACS. However, it is important to note that intra-rater reliability could have been included to strengthen the overall conclusions of this study. Lastly, for our subgroup analysis based on years of experience, we established a cut-off point for experienced athletes at 5 years. This was based on previous research suggesting that 4-5 years of experience in sports results in cognitive, emotional, and physical developments [67]. However, different sports may require less or more years of experience for these cognitive, emotional, and physical changes to occur.

Previous research has raised questions about the predictive accuracy of screening tests for sports-related injuries [68,69]. It is well established that sports injuries result from a multifaceted interplay of factors, many of which are inherently unpredictable. Furthermore, variables such as an athlete’s strength exhibit significant diurnal variations. Studies have indicated that athletes may experience daily fluctuations in neuromuscular performance during a training season [70] and that the factors influencing performance can change from one training session to the next [70]. As such, it is essential to recognise that the cut-off points derived from prior prospective investigations should be used primarily to assess an athlete’s readiness for participation or to identify potential performance limitations, rather than to predict future injuries if these criteria are not met [67]. Given that the modified ACS was developed to efficiently assess the key anatomical regions associated with injury occurrence in overhead sports, the outcomes of this study should serve as a valuable resource for coaching staff when designing individualised components of injury prevention programs. This information can guide them in prioritising specific aspects during training sessions.

## 5. Conclusions

In conclusion, the modified ACS demonstrates promising discriminant validity in identifying significant limitations in range of motion (ROM) and balance among overhead athletes participating in various sports. Subgroup analysis based on gender, sport, and experience showed that our findings remained consistent. These limitations can potentially impact performance and increase the risk of injury by overloading other anatomical structures. With its high sensitivity, moderate to strong specificity, and associations with prevalent risk factors, the modified ACS proves to be a valuable tool for coaches and sports personnel. It enables them to assess motion limitations specific to sports and implement targeted prevention strategies effectively. The incorporation of the modified ACS into routine assessments has the potential to enhance injury prevention programs, thereby contributing to the long-term athletic performance and wellbeing of overhead athletes. Nevertheless, further research is warranted, particularly involving larger sample sizes and interventional studies, to solidify its effectiveness in enhancing athletic performance and preventing injuries. Overall, the modified ACS holds promise as an effective screening tool in overhead athletes.

## Figures and Tables

**Table 1:** ACS tests and the respective comparisons with the musculoskeletal risk factors.

ACS	Risk Factors
Reciprocal shoulder mobility	Limited shoulder internal rotation (IR) passive range of motion (PROM) <45°: The dominant shoulder demonstrated less than 45° of IR PROM at 90° of abduction [27]. Glenohumeral internal rotation deficit (GIRD) ≥20°: The difference between the non-dominant shoulder’s IR and the dominant shoulder’s IR was equal to or greater than 20° [10,28]. Shoulder total range of motion deficit (TROM) ≥10°: The TROM (total range of motion) difference between the dominant and non-dominant shoulders equalled or exceeded 10° [27,29]. Shoulder flexion deficit ≥5°: The difference between the dominant shoulder’s flexion PROM and the non-dominant shoulder’s flexion PROM was equal to or greater than 5° [30]. Thoracic spine rotation PROM <50°: Either the dominant or non-dominant thoracic rotation PROM measured below 50° when in a quadruped position [31,32]
Total Body Rotation	Limited hip internal rotation (IR) passive range of motion (PROM) ≤36°: Either the stance or stride hip showed an IR PROM of 36° or less when the participant was in the prone position [11,33]. Restricted hip external rotation (ER) passive range of motion (PROM) ≤40°: Either the stance or stride hip exhibited an ER PROM of 40° or less with the participant in the prone position [12].
Lower Body Diagonal Reach	Normalised Y-Balance test–posterior–lateral (YBT-PL) reach distance: The YBT-PL reach was evaluated for both the stance and stride legs using the YBT. To account for the potential influence of player height on reach distance, the YBT-PL reach was adjusted by dividing it by the length of the participant’s dominant lower limb and then multiplying the result by 100. The average values of the normalised YBT-PL reach distances were computed for each age group. Any YBT-PL reach distances falling below the lower third quartile for the respective age categories—youth (<92 cm), high school (<95 cm), and college (<98 cm)—were considered as risk factors [34]. YBT-PL reach asymmetry: The absolute difference between the YBT-PL reach distance when using the stance leg and the stride leg was equal to or greater than 5.5 cm [35].
Rotary Stability	Closed Kinetic Chain Upper Extremity Stability (CKCUES) test: Athletes that scored below the reported reference values (18.5 touches for males and 20.5 touches for females from a modified position) [25,26].

Abbreviations: Internal Rotation (IR); External Rotation (ER); Passive Range of Motion (PROM); Total Range of Motion (TROM); Closed Kinetic Chain Upper Extremity Stability (CKCUES); Y-Balance Test–Posterior–Lateral (YBT-PL); Glenohumeral Internal Rotation Deficit (GIRD).

**Table 2:** Demographics of the included sample (N = 62).

Mean Age	21.4
Gender (Male–Female)	26–36
Height (cm)	177.7 ± 10.8
Weight (kg)	72.6 ± 13.8
BMI	23
Limb dominance (L: Left, R: Right)	4 L–58 R
Experience (years)	7.7 ± 4.6
Sport	
Volleyball	36
Basketball	19
Tennis	7

**Table 3:** Detecting accuracy and association in each component of the modified ACS compared with the risk factors.

Reciprocal Shoulder Mobility			90/90 Total Body Rotation			Lower-Body Diagonal Reach			Core Stability		
	≥1 Risk Factor			≥1 Risk Factor			≥1 Risk Factor			≥1 Risk Factor	
Shoulder mobility	Yes	No	Total Body Rotation	Yes	No	Diagonal Reach	Yes	No	Rotary Stability	Yes	No
Fail	25	18	Fail	23	2	Fail	31	4	Fail	13	5
Pass	5	14	Pass	3	34	Pass	4	23	Pass	3	41
Chi-square for association	*p* = 0.032, phi = 0.273			*p* = 0.001, phi = 0.905			*p* = 0.001, phi = 0.704			*p* = 0.001, phi = 0.678	

**Table 4:** Sensitivity, specificity, positive predictive value (PPV), negative predictive value (NPV), likelihood ratios (LRs), and accuracy of the components of the modified ACS.

ACS Component	Reciprocal Shoulder Mobility		Total Body Rotation		Lower Body Diagonal Reach		Rotary Stability	
Statistic	Value	95% CI	Value	95% CI	Value	95% CI	Value	95% CI
Sensitivity	83.33%	65.28% to 94.36%	88.46%	69.85% to 97.55%	88.57%	73.26% to 96.80%	81.25%	54.35% to 95.95%
Specificity	43.75%	26.36% to 62.34%	94.44%	81.34% to 99.32%	85.19%	66.27% to 95.81%	89.13%	76.43% to 96.38%
Positive Likelihood Ratio	1.48	1.05 to 2.09	15.92	4.11 to 61.67	5.98	2.40 to 14.89	7.47	3.16 to 17.67
Negative Likelihood Ratio	0.38	0.16 to 0.93	0.12	0.04 to 0.36	0.13	0.05 to 0.34	0.21	0.08 to 0.59
Disease Prevalence	48.39%	35.50% to 61.44%	41.94%	29.51% to 55.15%	56.45%	43.26% to 69.01%	25.81%	15.53% to 38.50%
Positive Predictive Value	58.14%	49.59% to 66.23%	92.00%	74.81% to 97.80%	88.57%	75.68% to 95.07%	72.22%	52.38% to 86.01%
Negative Predictive Value	73.68%	53.45% to 87.23%	91.89%	79.58% to 97.05%	85.19%	69.29% to 93.61%	93.18%	83.06% to 97.44%
Accuracy	62.90%	49.69% to 74.84%	91.94%	82.17% to 97.33%	87.10%	76.15% to 94.26%	87.10%	76.15% to 94.26%
Odds Ratio	3.88	1.18 to 12.74	130.3	20.171 to 842.128	48.87	11.086 to 215.461	35.53	7.4562 to 169.338

**Table 5:** Inter-rater reliability of the modified ACS.

ACS Component	Reciprocal Shoulder Mobility		Total Body Rotation		Lower Body Diagonal Reach		Rotary Stability	
	Value	95% CI	Value	95% CI	Value	95% CI	Value	95% CI
Inter-rater reliability	0.85	0.71 to 0.98	0.86	0.74 to 0.97	0.90	0.80 to 0.99	0.88	0.76 to 0.99

## Data Availability

Available upon reasonable request by the primary author.

## Supplementary Materials

The following supporting information can be downloaded at: https://www.mdpi.com/article/10.3390/healthcare11233046/s1, Table S1. Sensitivity, specificity, positive predictive value (PPV), negative predictive value (NPV), likelihood ratios (LRs) and accuracy of the components of the modified ACS for Volleyball Athletes only. Table S2. Sensitivity, specificity, positive predictive value (PPV), negative predictive value (NPV), likelihood ratios (LRs) and accuracy of the components of the modified ACS for Basketball Athletes only. Table S3. Sensitivity, specificity, positive predictive value (PPV), negative predictive value (NPV), likelihood ratios (LRs) and accuracy of the components of the modified ACS for Tennis Athletes only. Table S4. Sensitivity, specificity, positive predictive value (PPV), negative predictive value (NPV), likelihood ratios (LRs) and accuracy of the components of the modified ACS for Male Athletes only. Table S5. Sensitivity, specificity, positive predictive value (PPV), negative predictive value (NPV), likelihood ratios (LRs) and accuracy of the components of the modified ACS for Female Athletes only. Table S6. Sensitivity, specificity, positive predictive value (PPV), negative predictive value (NPV), likelihood ratios (LRs) and accuracy of the components of the modified ACS for experienced athletes only (>5 years of experience in the sport). Table S7. Sensitivity, specificity, positive predictive value (PPV), negative predictive value (NPV), likelihood ratios (LRs) and accuracy of the components of the modified ACS for non-experienced athletes only (≤ 5 years of experience in the sport).
