# Supplementary material for: Preliminary Insights into the Diagnostic Accuracy of the Modified Arm Care Screen Test for Overhead Athletes: An On-Field Tool for Injury Prevention
Source: Healthcare (Basel). 2023 Nov 26;11(23):3046. doi: 10.3390/healthcare11233046 (PMC10705961; doi:10.3390/healthcare11233046)
Supplement: Supplementary file 1 [file healthcare-11-03046-s001.zip › healthcare-2704886-supplementary.pdf]

Table S1. Sensitivity, specificity, positive predictive value (PPV), negative predictive value (NPV), likelihood ratios (LRs) and accuracy of the components of the modified ACS for **Volleyball** Athletes only.

**ACS Component Volleyball Athletes**	**Reciprocal shoulder mobility**		**Total Body Rotation**		**Lower Body Diagonal Reach**		**Rotary Stability**	
**Statistic**	**Value**	**95% CI**	**Value**	**95% CI**	**Value**	**95% CI**	**Value**	**95% CI**
**Sensitivity**	94.44%	72.71% to 99.86%	100.00%	73.54% to 100.00%	100.00%	83.16% to 100.00%	100.00%	63.06% to 100.00%
**Specificity**	27.78%	9.69% to 53.48%	100.00%	85.75% to 100.00%	100.00%	75.29% to 100.00%	89.29%	71.77% to 97.73%
**Positive Likelihood Ratio**	1.31	0.96 to 1.78					9.33	3.20 to 27.19
**Negative Likelihood Ratio**	0.2	0.03 to 1.55	0		0		0	
**Disease prevalence**	50.00%	32.92% to 67.08%	33.33%	18.56% to 50.97%	60.61%	42.14% to 77.09%	22.22%	10.12% to 39.15%
**Positive Predictive Value**	56.67%	49.02% to 64.01%	100.00%	73.54% to 100.00%	100.00%	83.16% to 100.00%	72.73%	47.79% to 88.60%
**Negative Predictive Value**	83.33%	39.27% to 97.48%	100.00%	85.75% to 100.00%	100.00%	75.29% to 100.00%	100.00%	86.28% to 100.00%
**Accuracy**	61.11%	43.46% to 76.86%	100.00%	90.26% to 100.00%	100.00%	89.42% to 100.00%	91.67%	77.53% to 98.25%
**Odds ratio**	94.44%	72.71% to 99.86%	100.00%	73.54% to 100.00%	100.00%	83.16% to 100.00%	100.00%	63.06% to 100.00%

Table S2. Sensitivity, specificity, positive predictive value (PPV), negative predictive value (NPV), likelihood ratios (LRs) and accuracy of the components of the modified ACS for **Basketball** Athletes only.

**ACS Component Volleyball Athletes**	**Reciprocal shoulder mobility**		**Total Body Rotation**		**Lower Body Diagonal Reach**		**Rotary Stability**	
**Statistic**	**Value**	**95% CI**	**Value**	**95% CI**	**Value**	**95% CI**	**Value**	**95% CI**
**Sensitivity**	87.50%	47.35% to 99.68%	83.33%	51.59% to 97.91%	75.00%	42.81% to 94.51%	100.00%	47.82% to 100.00%
**Specificity**	63.64%	30.79% to 89.07%	100.00%	59.04% to 100.00%	100.00%	59.04% to 100.00%	100.00%	76.84% to 100.00%
**Positive Likelihood Ratio**	2.41	1.06 to 5.49						
**Negative Likelihood Ratio**	0.2	0.03 to 1.30	0.17	0.05 to 0.59	0.25	0.09 to 0.67	0	
**Disease prevalence**	42.11%	20.25% to 66.50%	63.16%	38.36% to 83.71%	63.16%	38.36% to 83.71%	26.32%	9.15% to 51.20%
**Positive Predictive Value**	63.64%	43.42% to 79.96%	100.00%	69.15% to 100.00%	100.00%	66.37% to 100.00%	100.00%	47.82% to 100.00%
**Negative Predictive Value**	87.50%	51.47% to 97.88%	77.78%	49.69% to 92.54%	70.00%	46.69% to 86.14%	100.00%	76.84% to 100.00%

**Accuracy**	73.68%	48.80% to 90.85%	89.47%	66.86% to 98.70%	84.21%	60.42% to 96.62%	100.00%	82.35% to 100.00%
**Odds ratio**	87.50%	47.35% to 99.68%	83.33%	51.59% to 97.91%	75.00%	42.81% to 94.51%	100.00%	47.82% to 100.00%

Table S3. Sensitivity, specificity, positive predictive value (PPV), negative predictive value (NPV), likelihood ratios (LRs) and accuracy of the components of the modified ACS for **Tennis** Athletes only.

**ACS Component Volleyball Athletes**	**Reciprocal shoulder mobility**		**Total Body Rotation**		**Lower Body Diagonal Reach**		**Rotary Stability**	
**Statistic**	**Value**	**95% CI**	**Value**	**95% CI**	**Value**	**95% CI**	**Value**	**95% CI**
**Sensitivity**	25.00%	0.63% to 80.59%	66.67%	9.43% to 99.16%	50.00%	6.76% to 93.24%	28.57%	3.67% to 70.96%
**Specificity**	66.67%	9.43% to 99.16%	100.00%	39.76% to 100.00%	100.00%	29.24% to 100.00%	100.00%	2.50% to 100.00%
**Positive Likelihood Ratio**	0.75	0.07 to 7.73						
**Negative Likelihood Ratio**	1.12	0.42 to 3.00	0.33	0.07 to 1.65	0.5	0.19 to 1.33	0.71	0.45 to 1.14
**Disease prevalence**	57.14%	18.41% to 90.10%	42.86%	9.90% to 81.59%	57.14%	18.41% to 90.10%	87.50%	47.35% to 99.68%
**Positive Predictive Value**	50.00%	8.84% to 91.16%	100.00%	15.81% to 100.00%	100.00%	15.81% to 100.00%	100.00%	15.81% to 100.00%
**Negative Predictive Value**	40.00%	20.01% to 63.98%	80.00%	44.67% to 95.20%	60.00%	36.02% to 79.99%	16.67%	11.13% to 24.22%
**Accuracy**	42.86%	9.90% to 81.59%	85.71%	42.13% to 99.64%	71.43%	29.04% to 96.33%	37.50%	8.52% to 75.51%
**Odds ratio**	25.00%	0.63% to 80.59%	66.67%	9.43% to 99.16%	50.00%	6.76% to 93.24%	28.57%	3.67% to 70.96%

Table S4. Sensitivity, specificity, positive predictive value (PPV), negative predictive value (NPV), likelihood ratios (LRs) and accuracy of the components of the modified ACS for **Male** Athletes only.

**ACS Component Volleyball Athletes**	**Reciprocal shoulder mobility**		**Total Body Rotation**		**Lower Body Diagonal Reach**		**Rotary Stability**	
**Statistic**	**Value**	**95% CI**	**Value**	**95% CI**	**Value**	**95% CI**	**Value**	**95% CI**
**Sensitivity**	83.33%	51.59% to 97.91%	87.50%	61.65% to 98.45%	81.25%	54.35% to 95.95%	87.50%	47.35% to 99.68%
**Specificity**	58.33%	27.67% to 84.83%	100.00%	69.15% to 100.00%	100.00%	63.06% to 100.00%	100.00%	80.49% to 100.00%
**Positive Likelihood Ratio**	2	0.98 to 4.09						
**Negative Likelihood Ratio**	0.29	0.07 to 1.10	0.12	0.03 to 0.46	0.19	0.07 to 0.52	0.12	0.02 to 0.78

**Disease prevalence**	50.00%	29.12% to 70.88%	61.54%	40.57% to 79.77%	66.67%	44.68% to 84.37%	32.00%	14.95% to 53.50%
**Positive Predictive Value**	66.67%	49.44% to 80.36%	100.00%	76.84% to 100.00%	100.00%	75.29% to 100.00%	100.00%	59.04% to 100.00%
**Negative Predictive Value**	77.78%	47.51% to 93.12%	83.33%	57.76% to 94.81%	72.73%	49.02% to 88.09%	94.44%	73.10% to 99.07%
**Accuracy**	70.83%	48.91% to 87.38%	92.31%	74.87% to 99.05%	87.50%	67.64% to 97.34%	96.00%	79.65% to 99.90%
**Odds ratio**	83.33%	51.59% to 97.91%	87.50%	61.65% to 98.45%	81.25%	54.35% to 95.95%	87.50%	47.35% to 99.68%

Table S5. Sensitivity, specificity, positive predictive value (PPV), negative predictive value (NPV), likelihood ratios (LRs) and accuracy of the components of the modified ACS for **Female** Athletes only.

**ACS Component Volleyball Athletes**	**Reciprocal shoulder mobility**		**Total Body Rotation**		**Lower Body Diagonal Reach**		**Rotary Stability**	
**Statistic**	**Value**	**95% CI**	**Value**	**95% CI**	**Value**	**95% CI**	**Value**	**95% CI**
**Sensitivity**	82.35%	56.57% to 96.20%	90.91%	58.72% to 99.77%	94.74%	73.97% to 99.87%	66.67%	29.93% to 92.51%
**Specificity**	36.84%	16.29% to 61.64%	100.00%	86.28% to 100.00%	81.25%	54.35% to 95.95%	88.00%	68.78% to 97.45%
**Positive Likelihood Ratio**	1.3	0.87 to 1.96			5.05	1.81 to 14.09	5.56	1.75 to 17.68
**Negative Likelihood Ratio**	0.48	0.15 to 1.56	0.09	0.01 to 0.59	0.06	0.01 to 0.44	0.38	0.15 to 0.97
**Disease prevalence**	47.22%	30.41% to 64.51%	30.56%	16.35% to 48.11%	54.29%	36.65% to 71.17%	26.47%	12.88% to 44.36%
**Positive Predictive Value**	53.85%	43.69% to 63.69%	100.00%	69.15% to 100.00%	85.71%	68.27% to 94.36%	66.67%	38.59% to 86.42%
**Negative Predictive Value**	70.00%	41.67% to 88.40%	96.15%	79.41% to 99.39%	92.86%	65.54% to 98.89%	88.00%	74.22% to 94.92%
**Accuracy**	58.33%	40.76% to 74.49%	97.22%	85.47% to 99.93%	88.57%	73.26% to 96.80%	82.35%	65.47% to 93.24%
**Odds ratio**	82.35%	56.57% to 96.20%	90.91%	58.72% to 99.77%	94.74%	73.97% to 99.87%	66.67%	29.93% to 92.51%

Table S6. Sensitivity, specificity, positive predictive value (PPV), negative predictive value (NPV), likelihood ratios (LRs) and accuracy of the components of the modified ACS for experienced athletes only (>5 years of experience in the sport).

**ACS Component Volleyball Athletes**	**Reciprocal shoulder mobility**		**Total Body Rotation**		**Lower Body Diagonal Reach**		**Rotary Stability**	
**Statistic**	**Value**	**95% CI**	**Value**	**95% CI**	**Value**	**95% CI**	**Value**	**95% CI**
**Sensitivity**	95.24%	76.18% to 99.88%	92.86%	66.13% to 99.82%	88.46%	69.85% to 97.55%	75.00%	42.81% to 94.51%

**Specificity**	52.17%	30.59% to 73.18%	100.00%	88.43% to 100.00%	94.12%	71.31% to 99.85%	90.00%	73.47% to 97.89%
**Positive Likelihood Ratio**	1.99	1.29 to 3.08			15.04	2.23 to 101.21	7.5	2.44 to 23.04
**Negative Likelihood Ratio**	0.09	0.01 to 0.64	0.07	0.01 to 0.47	0.12	0.04 to 0.36	0.28	0.10 to 0.75
**Disease prevalence**	47.73%	32.46% to 63.31%	31.82%	18.61% to 47.58%	60.47%	44.41% to 75.02%	28.57%	15.72% to 44.58%
**Positive Predictive Value**	64.52%	54.00% to 73.79%	100.00%	75.29% to 100.00%	95.83%	77.36% to 99.36%	75.00%	49.41% to 90.21%
**Negative Predictive Value**	92.31%	63.01% to 98.83%	96.77%	81.94% to 99.50%	84.21%	64.64% to 93.96%	90.00%	77.03% to 96.02%
**Accuracy**	72.73%	57.21% to 85.04%	97.73%	87.98% to 99.94%	90.70%	77.86% to 97.41%	85.71%	71.46% to 94.57%
**Odds ratio**	95.24%	76.18% to 99.88%	92.86%	66.13% to 99.82%	88.46%	69.85% to 97.55%	75.00%	42.81% to 94.51%

Table S7. Sensitivity, specificity, positive predictive value (PPV), negative predictive value (NPV), likelihood ratios (LRs) and accuracy of the components of the modified ACS for non-experienced athletes only (≤ 5 years of experience in the sport).

**ACS Component Volleyball Athletes**	**Reciprocal shoulder mobility**		**Total Body Rotation**		**Lower Body Diagonal Reach**		**Rotary Stability**	
**Statistic**	**Value**	**95% CI**	**Value**	**95% CI**	**Value**	**95% CI**	**Value**	**95% CI**
**Sensitivity**	55.56%	21.20% to 86.30%	91.67%	61.52% to 99.79%	80.00%	44.39% to 97.48%	66.67%	9.43% to 99.16%
**Specificity**	22.22%	2.81% to 60.01%	46.15%	19.22% to 74.87%	100.00%	59.04% to 100.00%	100.00%	63.06% to 100.00%
**Positive Likelihood Ratio**	0.71	0.36 to 1.41	1.7	1.00 to 2.90				
**Negative Likelihood Ratio**	2	0.48 to 8.31	0.18	0.03 to 1.29	0.2	0.06 to 0.69	0.33	0.07 to 1.65
**Disease prevalence**	50.00%	26.02% to 73.98%	48.00%	27.80% to 68.69%	58.82%	32.92% to 81.56%	27.27%	6.02% to 60.97%
**Positive Predictive Value**	41.67%	26.56% to 58.52%	61.11%	48.02% to 72.78%	100.00%	63.06% to 100.00%	100.00%	15.81% to 100.00%
**Negative Predictive Value**	33.33%	10.75% to 67.50%	85.71%	45.65% to 97.72%	77.78%	50.33% to 92.36%	88.89%	61.75% to 97.54%
**Accuracy**	38.89%	17.30% to 64.25%	68.00%	46.50% to 85.05%	88.24%	63.56% to 98.54%	90.91%	58.72% to 99.77%
**Odds ratio**	55.56%	21.20% to 86.30%	91.67%	61.52% to 99.79%	80.00%	44.39% to 97.48%	66.67%	9.43% to 99.16%
